# Plasticity in *EGFR*-mutant NSCLC: SCLC transformation and subsequent re-emergence: case report

**DOI:** 10.3389/fonc.2026.1902531

**Published:** 2026-07-09

**Authors:** Farzaneh Atashrazm, Gabrielle Bradshaw, Jonathan Clark, Sagun Parakh

**Affiliations:** 1 Department of Medical Oncology, Austin Health, Melbourne, VIC, Australia; 2 Olivia Newton-John Cancer Research Institute, Melbourne, VIC, Australia; 3 Department of Pathology, Austin Health, Melbourne, VIC, Australia

**Keywords:** *EGFR* mutant non-small cell lung cancer, re-biopsy, re-emergence, small cell transformation, tumor plasticity

## Abstract

Small cell lung cancer (SCLC) transformation is a recognized resistance mechanism in *EGFR*-mutated non-small cell lung cancer (NSCLC) following treatment with tyrosine kinase inhibitors (TKIs). However, subsequent re-emergence of adenocarcinoma following SCLC transformation is exceedingly rare and remains poorly understood. We report a case of metastatic *EGFR* L858R-mutant lung adenocarcinoma that underwent transformation to small cell lung cancer (SCLC) and subsequently re-emerged as adenocarcinoma with a distinct and complex resistance profile. This case highlights the extreme lineage plasticity of *EGFR*-mutant NSCLC and underscores the necessity of serial biopsies to identify evolving distinct clonal populations and guide therapy.

## 1 Introduction

Small cell lung cancer (SCLC) transformation represents a rare mechanism of resistance to EGFR-tyrosine kinase inhibitors (TKIs) in *EGFR*-mutated non-small cell lung cancer (NSCLC), with an incidence of 5-15% (1). The precise mechanism behind this transformation is not yet completely understood, however, mechanisms proposed include epithelial-to-mesenchymal transition (EMT); mutations to signaling pathways such as RB1/TP53 and microenvironmental changes (2).

While transformation from adenocarcinoma to SCLC is a recognized mechanism, subsequent re-acquisition of an adenocarcinoma phenotype following prior SCLC transformation (herein termed “adenocarcinoma re-emergence”) has rarely been described, limiting current understanding of its biological and clinical significance. This phenomenon may reflect dynamic clonal evolution and the coexistence of distinct tumor populations that are differentially selected during treatment. Consequently, the molecular features and therapeutic implications of adenocarcinoma re-emergence remain largely undefined.

We report a unique case of bidirectional lineage plasticity characterized by initial transformation to SCLC followed by a return to an adenocarcinoma phenotype harboring novel and complex EGFR resistance mutations.

## 2 Case presentation

A 79-year-old male former smoker with a 20 pack-year history presented with exertional dyspnea and was found to have bilateral cavitating pulmonary nodules on computed tomography (CT) of the chest. His medical history was significant for prostate cancer treated with radical prostatectomy, hypertension managed with amlodipine, and prior stroke without residual neurological deficits. The patient had no family history of malignancy. The patient’s Eastern Cooperative Oncology Group (ECOG) performance status at diagnosis was 1. Whole-body ^18^F-fluorodeoxyglucose (FDG) positron emission tomography (PET) demonstrated FDG-avid lesions in both lungs, lymph nodes, and bone. CT of the brain showed no intracranial metastases. Histopathological examination of a supraclavicular lymph node core biopsy confirmed lung adenocarcinoma (**Figure 1**; **Supplementary Material**). Next-generation sequencing (NGS) identified *EGFR* L858R and *TP53* G266* mutations and a *RET* duplication with a relative copy number of 2.62. Notably, no mutations in *RB1* or *PIK3CA* were detected (**Supplementary Material**). The patient commenced monotherapy with the third-generation EGFR tyrosine kinase inhibitor (TKI), osimertinib, which was well-tolerated, with only grade 1 skin dryness reported. The best clinical response was a partial response. After 11 months, restaging scans demonstrated progression in the left upper lobe of the lung and new bone metastases. Endobronchial ultrasound (EBUS)-guided transbronchial biopsy confirmed histopathology transformation to SCLC with immunohistochemical stains demonstrating the tumor cells were positive for CD56, synaptophysin and AE1/3 and negative for chromogranin and p40. The ki67 index was almost 100%, consistent with small cell carcinoma. NGS demonstrated persistence of the original *EGFR* L858R and *TP53* G266* with additional *RB1* splice site variant, *PIK3CA* E542K, low level *MYC* amplification, and extensive copy number alterations (**Table 1**). Consequently, osimertinib was discontinued, and platinum-doublet chemotherapy (carboplatin (AUC 5) D1 and etoposide (100mg/m^2^) D1-3) with atezolizumab 1200mg intravenously every 3 weeks was commenced. He completed four cycles of induction therapy, achieving a partial response, and transitioned to maintenance atezolizumab 1200mg intravenously every 3 weeks.

**Figure 1:**
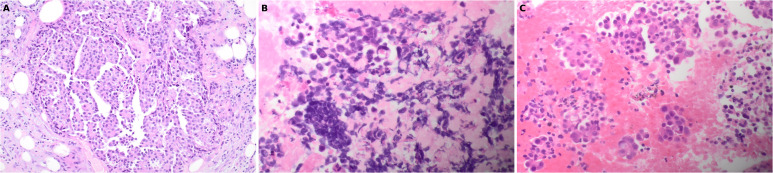
H&E-stained tissue sections demonstrating the histological evolution of the tumor: **(A)** initial adenocarcinoma from a right supraclavicular lymph node biopsy (20× magnification); **(B)** SCLC transformation identified on left upper lobe brushing (40× magnification); and **(C)** NSCLC re-emergence in a station 4R lymph node (40× magnification).

**Table 1:** Clinical, histologic, and genomic evolution over time.

Month	Event	Histology	Molecular profile	Therapy
0	Diagnosis	NSCLC (Adeno)	TP 70% **Driver:** *EGFR* L858R (VAF 29%) **Tumor suppressor***: TP53* G266* (VAF 43%) **CNVs:** *RET* duplication (CN: 2.62) *TP53* deletion: (CN: 1.48)	Osimertinib → Best response: Partial response
11	Progression	SCLC	TP 70% **Driver:** *EGFR* L858R (VAF 62%) **Tumor suppressor:** *TP53* G266* (VAF 91%), *RB1* c.1216-1G>T, p.? (VAF 89%) **Oncogenic co-alteration***: PIK3CA* E542K (VAF 40%) **CNVs:** *RET* deletion (CN:1.46) *RB1* deletion (CN:1.45), *EGFR* deletion (CN:1.56), *TP53* deletion (CN:1.31), *MYC* duplication (CN: 3.34), *ERBB2* duplication (CN: 2.61), *PIK3CA* duplication (CN: 2.92)	Platinum doublet chemotherapy + Atezolizumab →Maintenance Atezolizumab Best response: Partial response
16	Oligoprogression	SCLC		Stereotactic Radiotherapy (48 Gy in 4 fractions) + continued maintenance Atezolizumab
21	Multi-site progression	NSCLC (Adeno)	TP 80% **Driver:** *EGFR* L858R (VAF 22%) **Resistance***: EGFR* L718V (VAF 8.5%) **Tumor suppressor***: TP53* G266* (VAF 58%) **CNVs:** *EGFR* duplication (CN: 2.30), *RET* duplication (CN: 2.73), *TP53* deletion (CN:1.38)	Carboplatin + Paclitaxel → Osimertinib re-challenge Best response: Partial response

NSCLC, non-small cell lung cancer; SCLC, small cell lung cancer; CNVs, copy number variations; CN, copy number; TP, tumor purity; VAF, variant allele frequency.

Driver denotes the primary putative oncogenic driver alteration; Resistance denotes a putative acquired resistance alteration; Tumour suppressor denotes a pathogenic alteration in a tumour suppressor gene; Oncogenic co-alteration denotes an additional likely oncogenic alteration not considered the primary driver.

A restaging scan after 11 cycles of maintenance atezolizumab showed oligoprogression in the left upper lobe, which was treated with stereotactic radiotherapy (48 Gy in four fractions). The patient continued maintenance atezolizumab for a further five months without significant immune-mediated adverse events (irAEs). Subsequent restaging demonstrated multi-site progression in the lungs, thoracic lymph nodes, and bone (**Figure 2A**). Repeat EBUS biopsy revealed re-emergence of lung adenocarcinoma. Repeat NGS demonstrated a molecular profile similar to the original biopsy with additional *EGFR* copy number gain (2.30) and acquired *EGFR* L718V mutation. Notably, the *RB1*, *MYC*, and *PIK3CA* variants were no longer present. Given the recent exposure to checkpoint inhibitor, the patient commenced 3-weekly carboplatin (AUC 5) and paclitaxel 175mg/m^2^; however, treatment ceased after three cycles due to significant fatigue, after which osimertinib was recommenced. Four weeks following osimertinib re-commencement, PET imaging demonstrated a significant but incomplete metabolic response to therapy (**Figure 2B**).

**Figure 2:**
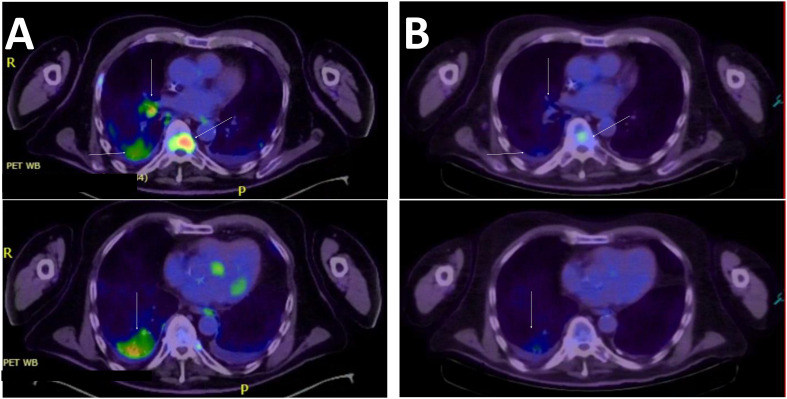
FDG-PET scan demonstrating disease status and treatment response. **A)** Timepoint: Transformed SCLC during maintenance atezolizumab, with FDG-avid lesions in the right lower lobe, T6 vertebra, and right hilar lymph node.; and **(B)** Timepoint: NSCLC re-emergence after three cycles of platinum-doublet chemotherapy and four weeks of osimertinib, with the corresponding lesions demonstrating a significant metabolic response.

## 3 Discussion

This case highlights the bidirectional plasticity and clonal selection of *EGFR*-mutant NSCLC and the therapeutic challenges posed by complex resistance mechanisms. While SCLC-transformation is a well-recognized mechanism of resistance to third-generation EGFR TKIs, subsequent re-emergence from SCLC back to adenocarcinoma is extremely rare, with only a single prior case reported (3). The rarity may partly reflect that re-biopsy at progression is not routinely performed, leading to potential under-recognition of such events.

*EGFR* mutant tumors with acquired concurrent *TP53* and *RB1* inactivation are more prone to SCLC transformation. In this case, biallelic *RB1* inactivation (splice site variant and loss of heterozygosity) emerged at the time of SCLC transformation, alongside persistent biallelic *TP53* inactivation, supporting a canonical transformation pathway. Together, these alterations likely enabled histologic transformation under therapeutic pressure.

A consistent finding across all three timepoints was persistence of the founding *EGFR* L858R driver and truncal *TP5*3 mutation (TP53 G266*), supporting a stable ancestral clone throughout disease evolution. Copy number loss of *TP53* was also present, consistent with biallelic inactivation, functional loss of tumor suppressor activity, and ongoing genomic instability, facilitating clonal diversification and phenotypic plasticity. TP53 alterations are also associated with poorer responses to TKI therapy in EGFR-mutant adenocarcinoma, further supporting their role in aggressive tumor biology (4). Chemotherapy has been shown to inhibit the development of EGFR-TKI–resistant clones, block the activation of bypass signaling pathways, and suppress resistance mutations (5). This activity likely explains why the patient achieved a partial response upon being retreated with osimertinib post chemotherapy despite the presence of EGFR L718V, which has shown to mediate osimertinib resistance. The rationale for rechallenging with osimertinib was the persistence of the founding EGFR L858R driver mutation, which remained the primary oncogenic driver of the disease.

The baseline adenocarcinoma harbored a RET copy number gain, which shifted to relative copy number loss at SCLC transformation before reappearing with adenocarcinoma recurrence after platinum-based chemotherapy. Dynamic changes in RET copy number further support a model of treatment-driven clonal selection. Relative RET copy number loss at SCLC transformation is consistent with the frequent chromosome 10q deletions reported in SCLC (6), while reappearance of RET copy number gain with adenocarcinoma recurrence likely reflects re-expansion of the original adenocarcinoma clone following chemotherapy. Similar tumor purity across all three specimens makes a technical explanation less likely.

Additional alterations in PIK3CA/PTEN/AKT pathway have been implicated in SCLC transformation of EGFR-mutant NSCLC (7). Consistent with this, at SCLC transformation our patient harbored an activating *PIK3CA* E542K mutation with concurrent *PIK3CA* copy number gain, supporting pathway activation. This occurred alongside extensive copy number alterations, reflecting genomic instability and clonal evolution associated with therapeutic resistance and histologic transformation. Unfortunately, longitudinal circulating DNA (ctDNA) analysis was not feasible for this patient, despite its emergence as an informative tool for detecting resistance mechanisms and monitoring therapeutic response in advanced NSCLC (8, 9) analysis would have enabled the capture of evolutionary trajectories among subclones contributing to tumor relapse and transformation. Furthermore, clonal tracking based on the variant allele frequency (VAF) of mutations could reveal how the subclonal architecture of the tumor evolves under the selective pressure exerted by sequential therapies.

Optimal management of transformed SCLC remains uncertain, with evidence limited to retrospective studies and case reports. These studies have shown responses to platinum/etoposide chemotherapy either alone or in combination with EGFR TKIs or immunotherapy and bevacizumab (10–12). Recent studies have shown no clear difference in overall survival between these different treatment strategies suggesting inclusion of immunotherapy or an EGFR-TKI may not provide value beyond chemotherapy (13, 14). In this case, NGS demonstrated persistence of the initial EGFR mutation after transformation, however EGFR expression has been reported to be downregulated in transformed SCLC, leading to reduced sensitivity to TKI therapy (10). The most striking feature of the second adenocarcinoma phase was the emergence of a complex *EGFR* profile (L718V mutation and a gain in *EGFR* copy number), both of which typically confer primary resistance to first- and third-generation TKIs. In this patient, due to the risk of immune-related adverse events with PD-(L)1 blockade and osimertinib, upfront chemotherapy was administered, which would also serve to treat the resistant sub-clonal populations prior to commencing osimertinib.

## 4 Conclusion

With the increasing use of third generation EGFR TKIs in the metastatic and adjuvant setting, this case emphasizes the necessity of repeat biopsies at each point of disease progression to guide therapy. The transition from a neuroendocrine phenotype back to an epithelial phenotype, accompanied by the emergence of a new *EGFR* resistance mutation, supports the evidence of dynamic clonal evolution, whereby distinct tumor subclones are suppressed or selected under the selective pressure of specific systemic therapies.

## 5 Patient perspectives

The patient provided informed consent for the publication of this case report. Throughout his diagnosis and treatment, he maintained a positive attitude. Although the need for repeat biopsies caused him significant anxiety, he fully understood their necessity to guide his care. He remains profoundly grateful for the multidisciplinary care he has received.

## Data Availability

The original contributions presented in the study are included in the article/**Supplementary Material**. Further inquiries can be directed to the corresponding author.
