# Supplementary material for: Plasticity in EGFR-mutant NSCLC: SCLC transformation and subsequent re-emergence: case report
Source: Front Oncol. 2026 Jul 9;16:1902531. doi: 10.3389/fonc.2026.1902531 (PMC13391332; doi:10.3389/fonc.2026.1902531)
Supplement: Supplementary file 1 [file DataSheet1.pdf]

A. Sequencing Method and Variant Annotation:

Genomic DNA isolated from tumour enriched FFPE tissue was analysed using an Agilent SureSelect custom-designed next generation sequencing (NGS) targeted gene panel for detection of single nucleotide variants, deletion/insertion variants, copy number variants and DNA fusions. The panel utilises SureSelect XTHS2 chemistry (hybridisation capture), including molecular barcodes, allowing multiplex data analysis, read deduplication and accurate variant calling. Automated library preparation was performed on the Agilent Magnis NGS Prep System. Library quantity and quality was assessed using a High Sensitivity D1000 ScreenTape assay on the Agilent 4150 TapeStation system. Indexed and pooled libraries were sequenced using Illumina NextSeq 1000/2000 (P1 XLEAP-SBS Reagent Kit 300 cycles). Sequencing data were processed using DRAGEN Enrichment v4.3.13 against the hg38 human reference genome, Genomical version 5.0.2 and variant annotation software Franklin by Qiagen, followed by variant visualisation in Integrative Genomics Viewer (IGV, Broad Institute).

Variants were further curated based on ACMG guidelines (Richard et al., 2015), AMP guidelines (Li et al., 2017) and the ClinGen/CGC/VICC classification of oncogenicity of somatic variants in cancer guidelines (Horak et al., 2022). Oncogenic/pathogenic and likely oncogenic/pathogenic variants in targeted regions were reported as per HGVS nomenclature version 21.1. Variants of uncertain significance (VUS) were not reported. Known polymorphisms and recurrent variants in the population were not reported. The assay did not distinguish between germline and somatic variants.

The mean target depth of the panel was 650x with a variant calling threshold of 200x with at least 8 mutant reads. The overall lower limit of detection for variant allele frequency (VAF) was 2.5%. The lower limit of detection for tumour purity for copy number duplications was 10%, and for copy number deletions was 65%. Copy number variants detected with more than two copies were called as duplications. Copy number variants detected with less than two copies were called as deletions.

Lung NGS Panel Coverage:

ALK (NM_004304.4: exons 20-25), *BAP1* (NM_004656.4: exons 1-17), BRAF (NM_004333.6: exons 8, 11-15), *CDKN2A* (NM_000077.5: exons 1-3), *CDKN2B* (NM_004936.4: exons 1-2), *EGFR* (NM_005228.5: exons 2, 3, 6-9, 15, 18-21), *ERBB2* (NM_004448.3: exons 10, 19-21, 24), *GNAS* (NM_00516.5: Exons 6-9), *HRAS* (NM_005343.4: exons 2-3), *KEAP1* (NM_203500.2: exons 2-6), *KRAS* (NM_004985.5: exons 2-4), *MAP2K1* (NM_002755.4: exons 2, 3, 5-7), *MET* (NM_000245.3: exons 2, 11, 14, 16, 19, 21), *MTAP* (NM_002451.4: exons 1-8), *MYC* (NM_002467.6: CNVs only), *NF1* (NM_001042492.3: exons 1-58), *NF2* (NM_000268.4: exons 1-16), *NRAS* (NM_002524.5: exons 2-5), *PIK3CA* (NM_006218.4: exons 2, 5, 7, 8, 10, 12, 14, 19-21), *PTPN11* (NM_002834.5: exons 3, 4, 7, 8, 12, 13), *RB1* (NM_000321.3: exons 1-27), *RET* (NM_020975.6: exons 5,8, 10, 11, 13-16), *ROS1* (NM_001378902.1: exons 31-42), *SMARCA4* (NM_003072.5: exons 2-36), *STK11* (NM_000455.5: exons 1-9), *TERT* (NM_198253.2: promoter region only), *TP53* (NM_000546.6: exons 1 (incl UTR), 2-11).

Fusions: *ALK* (NM_004304.4), *RET* (NM_020975.6), *ROS1* (NM_001378902.1).

B. **Histopathology findings:**

Initial NSCLC:

Specimen: R superclavicular lymph node biopsy.

Microscopic description: The sections show cores of fibroadipose tissue and skeletal muscle, with infiltration by adenocarcinoma comprising small nests and micropapillary structure formed by atypical epithelial cells with the moderate nuclear enlargement, small nucleoli and abundant, pale eosinophilic cytoplasm with scattered foci suggestive of intranuclear cytoplasmic mucin vacuoles. I

Immunohistochemistry: Positive for TTF-1.

Conclusion: Adenocarcinoma with micropapillary predominant architecture.

SCLC Transformation:

Specimen: 1. "left upper lobe brushing", 2. General wash.

Microscopic description: The sections show fragments of partially crushed tumour comprising sheets of closely packed cells with high N:C ratios, nuclear moulding, finely granular chromatin, inconspicuous nucleoli and small amounts of cytoplasm. Frequent fragments of apoptotic debris are present and there are foci of tumour cell necrosis.

Immunohistochemistry: The tumour cells are positive for CD56, synaptophysin, TTF-1 and AE1/3 and negative for chromogranin and p40. The ki67 index is close to 100%.

Conclusion: Small cell carcinoma.

NSCLC re-emergence:

Specimen: EBUS specimen `st 4R' and general bronchial washing.

Microscopic description: Preparations show variably sized groups of atypical cells with increased nuclear to cytoplasmic ratio and eccentrically placed nuclei and rare intracytoplasmic vacuoles.

Immunohistochemistry: TTF1(8G7G3/1)+ and PD-L1 negative (<1% of tumour cells).

Conclusion: Non-small cell carcinoma, favor adenocarcinoma.
